# Retrieval of Knotted and Stripped Guidewires Lost During Percutaneous Intervention on Kidney—A Case Series and Review of Literature

**DOI:** 10.5152/tud.2025.24159

**Published:** 2025-06-24

**Authors:** Sajad Ahmad Para, Sajad Ahmad Malik, Prince Muzafer Wani, Arif Hamid, Abdul Rouf Khawaja, Saqib Mehdi

**Affiliations:** 1 Department of Urology, Sher-i-Kashmir Institute of Medical Sciences, Srinagar, India; 2 Department of Urology, Government Medical College, Srinagar, India

**Keywords:** Knotted guidewire, percutaneous nephrolithotomy, urological complications

## Abstract

**Objective:**

Thecomplication associated with percutaneous interventions done on kidneys are usually easily identified and managed with defined protocols. These usually include bleeding, surrounding organ injury, or sepsis. Stripping or knotting of glidewire during these procedures is rare and have no defined management guidelines. There is a paucity in the literature about the management of these complications. A case series is reported on the management of this rare complication.

**Methods:**

A case series of 3 patients is presented, with 1 patient experiencing stripping of the glidewire and the other 2 having knotting of glidewire that got stuck during the percutaneous intervention done on the kidney. The guidewires were removed percutaneously with fluoroscopic guidance, thus avoiding the more morbid procedures of open surgery.

## Introduction

Nephrolithiasis is a common urological ailment with prevalence varying according to geographical location. In India, the prevalence of kidney stones is about 12% and increases to about 15% in the northern region. Percutaneous nephrolithotomy (PCNL) has been recommended as the standard procedure by the American Urological Association for treating large size kidney stones.^1^ Besides nephrolithiasis, percutaneous interventions on kidneys have been on rise to deal with pathology in and around the kidney. With advancements in optics, real-time fluoroscopy, and the innovation of better lithotripters, PCNL has been refined over the past few decades to achieve maximum stone clearance and minimize collateral damage.^2^ It is typically a safe procedure, but complications can occur even in best hands.^3^ The rate of complications following PCNL ranges from 15% to 25%, with most being minor ones. The most common complications include fever, urine leak, sepsis, surrounding organ injury, bleeding, or hemothorax.^4^ All these complications are usually easy to recognize and have defined protocols developed over a period of time to rectify them. A case series is presented on the rare complication of fragmentation, stripping, and knotting of guidewires during percutaneous procedures on kidneys, along with minimally invasive methods to deal with these rare complications.

## Material and Methods 

This study has been approved by the ethical committee of our institute, Sher-I-Kashmir institute of medical sciences with reference no. IEC/OA/32/2024. Written informed consent was obtained from all patients.

### Case 1:

**Figure 1.**
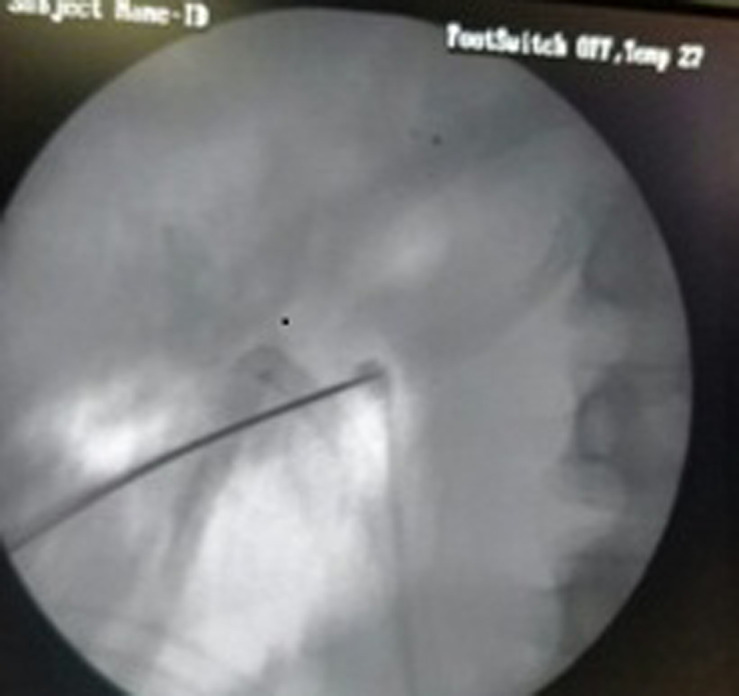
A puncture was made directly on the stripped loop.

**Figure 2.**
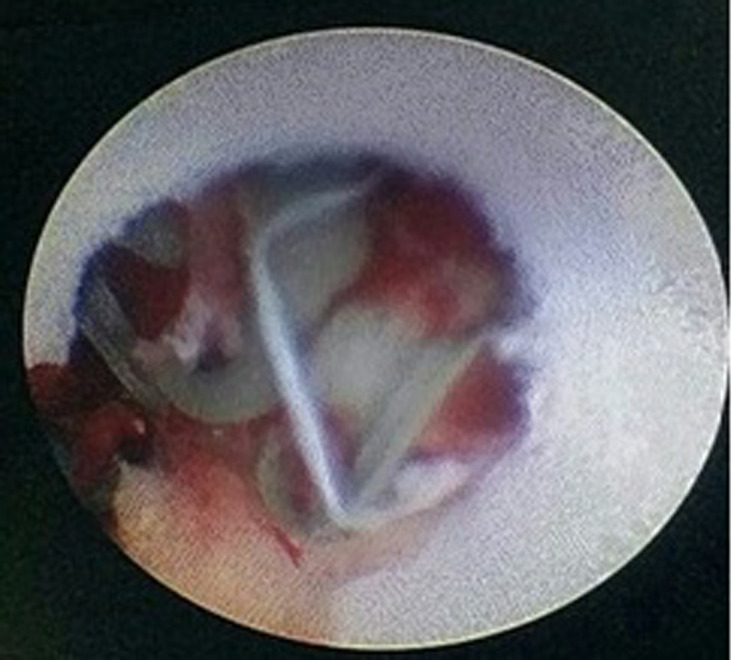
Endoscopic view of stripped loop.

A 32-year-old male underwent mini-PCNL (mPCNL) for a partial staghorn calculus extending into the middle calyx. Under fluoroscopic guidance, a middle calyceal puncture was made with an 18G initial puncture needle and a 0.032 inch hydrophilic guidewire was passed through. The guidewire could not be satisfactorily parked in the pelvicalyceal system (PCS) due to an impacted middle calyceal calculus and was getting coiled abnormally around the stone. After repeated failed attempts to pass the guidewire into the collecting system, it was removed with the puncture needle in place. During removal, the guidewire got stripped and the jacket was lost in the renal parenchyma. Another calyceal puncture was made and tract dilated to clear the stone. There was no endoscopic trace of the stripped guidewire in the PCS, though it was visible on fluoroscopy. The procedure was carried under spinal anesthesia and a lot of time was spent on searching for the stripped glidewire, so a DJ stent and nephrostomy tube were placed, and the procedure was concluded. A postoperative x-ray showed the stripped glidewire coiled in the renal area. A non-contrast computed tomography (CT) scan revealed the stripped glidewire in the renal parenchyma near the middle calyx. After 48 hours patient was again taken to operating room to remove the residual guidewire. Under fluoroscopic guidance the puncture was made directly on the stripped loop (Figure 1). A new guidewire was placed into the PCS under fluoroscopic guidance. Tract was dilated to 12 Fr and 8.5 Fr ureteroscope was passed through the sheath. The stripped wire was identified and removed intact (Figure 2).

### Case 2:

**Figure 3.**
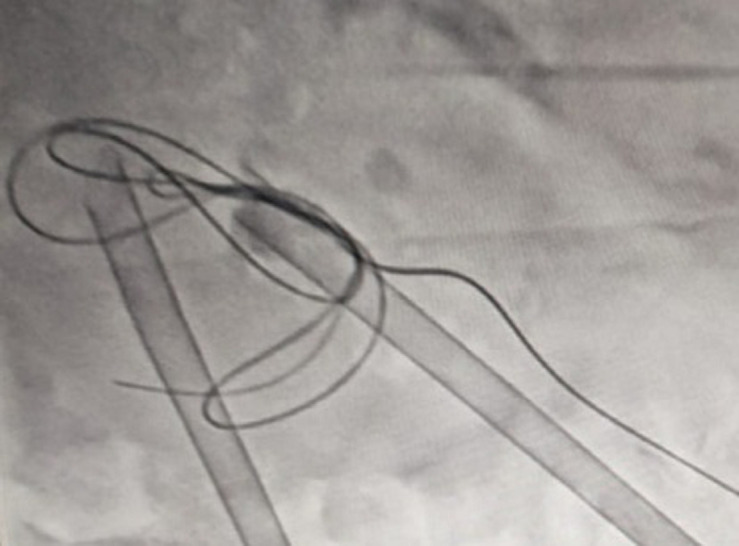
Knotted guidewire is seen on fluoroscopy with the Amplatz sheath in the middle and lower calyx.

**Figure 4.**
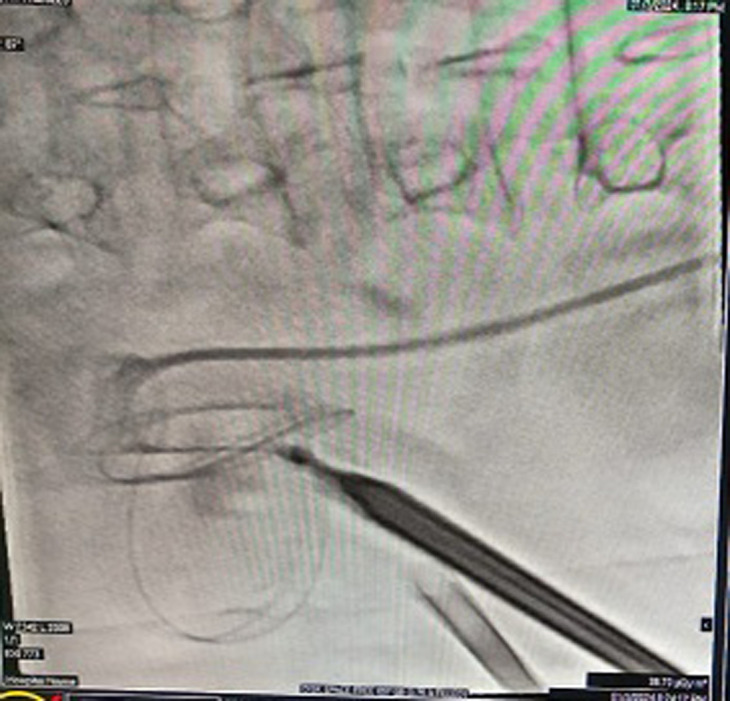
Tract dilated directly on the knotted guidewire.

A 52-year-old male with staghorn calculus was planned for mPCNL. An inferior calyceal puncture was made, and upon parking the guidewire, knotting occurred, and guide wire could not be removed after repeated failed attempts. The needle was removed, and guidewire left in place. A middle calyceal puncture was made and tract dilated to clear the stone from the middle calyx and pelvis. There was no endoscopic trace of the knotted guidewire. Another lower calyceal tract was established to remove the residual stone and search for the knotted guidewire. Again, there was no trace of the guidewire in PCS (Figure 3). To remove the knotted guidewire, the tract was dilated on the wire itself up to the knot, and 16 Fr Amplatz sheath was placed (Figure 4). Nephroscope was introduced and the knot divided with thulium fiber laser (TFL). All fragments were removed, and clearance confirmed on fluoroscopy.

#### Case 3:

**Figure 5.**
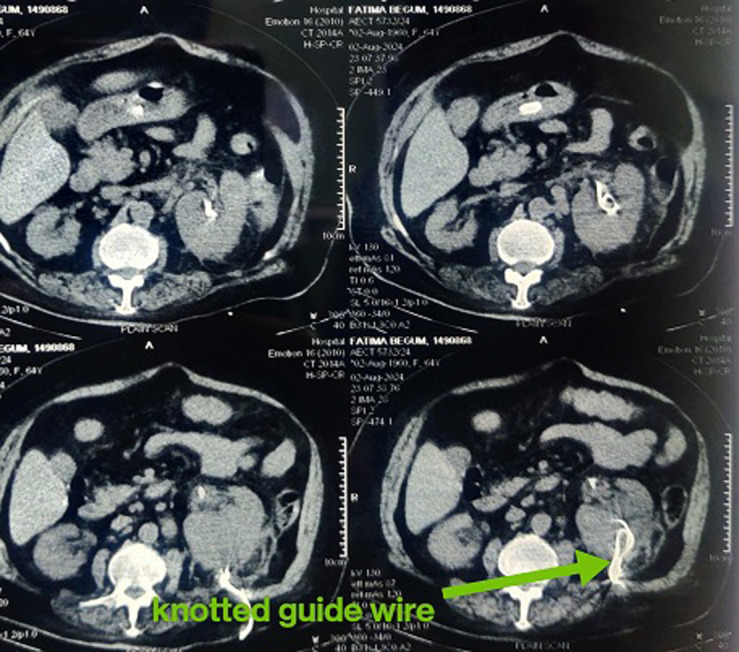
CT scan showing knotted guidewire partly inside renal parenchyma.

**Figure 6.**
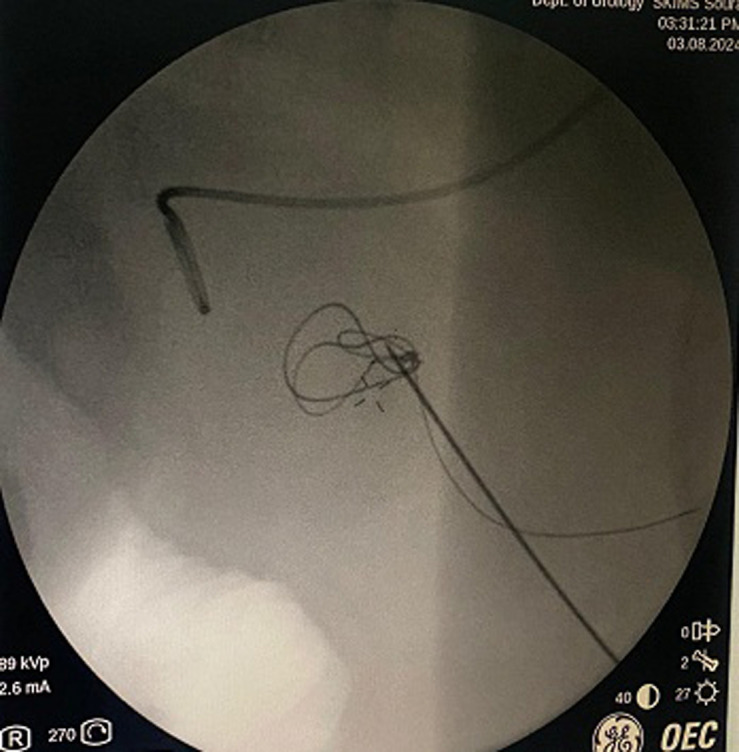
The knot was directly punctured under fluoroscopic guidance.

**Figure 7.**
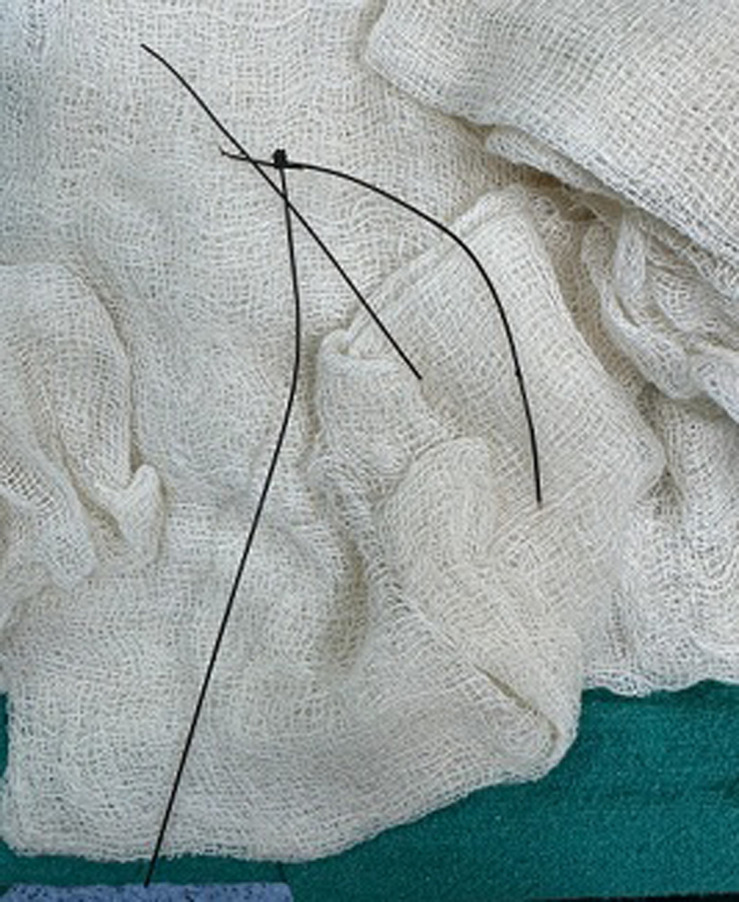
Knotted guide retrieved with a tight knot.

A 59-year-old female, hypertensive and diabetic, was admitted with urosepsis and deranged renal function. On evaluation, ultrasound (USG) examination revealed left-sided hydronephrosis with perinephric collection. A non-contrast CT showed left-sided hydroureteronephrosis with a 5 × 6 cm perinephric collection. A left-sided DJ stent was placed, and USG-guided drainage of the perinephric collection was planned. During malecot tube deployment, the guidewire could not be removed. The tube was taken out expecting the guidewire to accompany it, but the guidewire was knotted. Repeated attempts to remove the knotted guidewire resulted in breakage, leaving small stump outside the skin. A fresh malecot catheter was placed in the perinephric collection, and the fragmented guidewire was left in place and secured to skin to avoid internal migration. The patient improved, and a repeat CT showed no residual collection and the guidewire knotted outside the kidney in retroperitoneum (Figure 5). Three weeks later, the patient was planned for removal of the fragmented guidewire. Due to the small stump of the knotted guidewire left outside the skin, tract dilatation directly on the fragmented wire was not possible. Under fluoroscopic guidance, the knot was precisely punctured with an 18G initial puncture needle, and a hydrophilic guidewire was passed close to the knot. Tract was dilated up to 16 Fr, and an Amplatz sheath was placed (Figure 6). Nephroscope was introduced and knot was fragmented with Holmium:YAG laser. All fragments were removed, and the procedure was uneventful (Figure 7).

## Discussion

The PCNL qualifies as a standard treatment for large renal calculi and those who fail other treatment modalities. The procedure has been modified since its inception in 1976 with a reduction of tract diameters and precision in establishing safe tract.^5^ Although it is a less invasive procedure, complications have been reported even among experts in the field. The commonly occurring complications of PCNL or other percutaneous procedures done on the kidney are bleeding, surrounding organ injury, PCS perforation, and sepsis. These complications are easy to identify and manage without much morbidity or mortality.^3^ Difficulty arises when an unusual complication occurs and the surgeon is not prepared for it. These unusual complications do not have defined guidelines for management, and there is always a paucity of literature regarding them. They usually test the patience, courage, and intellect of the surgeon. Rare complications of stripping and fragmentation of guidewires were encountered during percutaneous procedures done on the kidney. In Case 1, stripping of the guidewire was identified intraoperatively while examining the remnant guidewire. The stripped loop could not be retrieved immediately because a lot of time was already spent searching the stripped fragment, and the spinal anesthetic effect was weaning off. When an abnormally coiled guidewire is pulled forcibly through a puncture needle, the jacket may get stripped from the nitinol core by the edge of the puncture needle.^6^ Repeated use of the same guidewire leads to the loss of hydrophilic coating, making them prone to stripping. Using a deformed (bent) guidewire during a percutaneous procedure is another major risk factor, with the bent point acting as a lead point for stripping.^7^ The use of a beveled puncture needle instead of a diamond tip needle easily peels off the coating from guidewire.^8^ Besides these technical mishaps, manufacturing flaws in the guidewire itself can make it prone to stripping. The percutaneous method used to remove the stripped guidewire is new and innovative with no literature available regarding the same.

The Terumo hydrophilic guidewire used consists of a super-elastic nitinol alloy core for optimal performance and kink resistance. The tungsten-infused polyurethane jacket provides superior tip and wire radiopacity. Overuse of the same guidewire, deformed guidewires, excessive use of force to park the guidewire, and compact PCS with tightly packed stones are some main risk factors for knotting.^9^ Fragmentation may be the result of the direct impact of laser energy on wires in endourologic procedures. Fragmented guidewires lost in PCS act as a nidus for stone formation, non-resolving urinary tract infections, and chronic draining sinuses. Franklin L et al reported a recurrent stone formed on a fragmented guidewire lost in PCS from a previous intervention.^10^ Fragmentation and knotting of guidewires have been reported in endovascular interventions, and the literature about the same in urology practice is sparse. Knotted guidewires can be removed by open technique or percutaneously through small tracts. When sufficient length is available, the tract may be dilated up to the knot, and a laser used to unravel the knotted guidewire. When sufficient wire is not available for tract dilation, the knot can be safely targeted under fluoroscopic guidance, and the tract established to divide the knot. Lasers play an indispensable role to break guidewire knots with TFL and Ho:YAG being equally effective in performing the task.^11^

Repeated use of hydrophilic guidewires leads to loss of the hydrophilic coating and makes them prone to stripping. Beveled puncture needles are more traumatic to hydrophilic guidewires. When locked in the PCS, the guidewire should not be pulled out with force against resistance; rather, the whole assembly with the needle intact should be pulled out. Locked and stripped guidewires can be safely retrieved percutaneously using small sheaths and endoscopes. Lasers play an important role in breaking guidewire knots

## Data Availability

The data that support the findings of this study are available on request from the corresponding author.
